# The CDM-Net Project: The Development, Implementation and Evaluation of a Broadband-Based Network for Managing Chronic Disease

**DOI:** 10.1155/2012/453450

**Published:** 2012-02-09

**Authors:** Kay Jones, Trisha Dunning, Beth Costa, Kristine Fitzgerald, Akuh Adaji, Colin Chapman, Leon Piterman, Moira Paterson, Peter Schattner, John Catford

**Affiliations:** ^1^Department of General Practice, Monash University, Building 1, 270 Ferntree Gully Road, Notting Hill 3168, VIC, Australia; ^2^Geelong Hospital and Deakin University, Kitchener House, P.O. Box 281, Geelong 3220, VIC, Australia

## Abstract

*Background*. In Australia most chronic disease management is funded by Medicare Australia through General Practitioner Management Plans (GPMPs) and Team Care Arrangements (TCAs). Identified barriers may be reduced effectively using a broadband-based network known as the Chronic Disease Management Service (CDMS). *Aims*. To measure the uptake and adherence to CDMS, test CDMS, and assess the adherence of health providers and patients to GPMPs and TCAs generated through CDMS. *Methods*. A single cohort before and after study. *Results*. GPMPs and TCAs increased. There was no change to prescribed medicines or psychological quality of life. Attendance at allied health professionals increased, but decreased at pharmacies. Overall satisfaction with CDMS was high among GPs, allied health professionals, and patients. *Conclusion*. This study demonstrates proof of concept, but replication or continuation of the study is desirable to enable the impact of CDMS on diabetes outcomes to be determined.

## 1. Introduction

The Chronic Disease Management Network (CDM-Net) project developed, implemented, and evaluated a broadband-based network of health services known as the Chronic Disease Management Service (CDMS) for managing chronic disease, using type 2 diabetes mellitus (T2DM) as the test disease.

A number of electronic systems are currently being used to enhance diabetes care including diabetes registers, clinical decision support systems, and web-based management programs. In order to find studies that evaluated information technology-based interventions that assist GPs to care for patients with diabetes, it was necessary to search the international literature, which could indicate that there is a pressing need to use the technology available for use in general practice in Australia to provide the best care for patients [1].

T2DM is a chronic progressive disease associated with high morbidity and mortality rates and currently affects an estimated 1.7 million Australians [2, 3]. T2DM accounts for 85% to 90% of all diagnosed cases of diabetes with lifestyle factors such as inactivity, obesity, and calorie-dense diets contribute to the increasing prevalence of T2DM [2]. Key diabetes management targets include achieving and maintaining HbA_1c_ levels at less than 7%, normal lipids, preventing diabetes complications, appropriate self-care and optimal quality of life [4]. Management is generally undertaken by general practitioners (GPs) supported by an interdisciplinary health care team.

In Australia, most chronic disease management undertaken by GPs is underwritten by Medicare Australia [5], and for eligible allied health services such as chiropractors, diabetes educators, dietitians, podiatrists, and others, and under certain conditions, can claim under Medicare Items 10950 to 10970 [6]. GPs receive financial incentives to use structured chronic disease care plans through Medicare Enhanced Primary Care (EPC) Item numbers for General Practitioner Management Plans (GPMPs Item 721) and are encouraged to collaborate with allied health providers and other health care professionals when developing Team Care Arrangements (TCAs Item 723) [4, 7].

While a steady increase in the number of GPMPs and TCAs has been reported, less than 14% of patients with chronic disease have a GPMP and/or a TCA [8]. In addition, Medicare claims data suggest only one in five of these plans is regularly followed up and reviewed at the recommended frequency [9] even though research suggests that patients with chronic disease may be hospitalised less frequently if they receive optimal care [10]. Conversely, improved outcomes for patients with diabetes have been reported when GPMPs and TCAs are used [11, 12], partly because of the structured and systematic approach to care.

Barriers identified by GPs for not using GPMPs include time constraints and difficulty communicating with other health providers [13]. Some barriers such as communication could be reduced by effectively using information technology (IT), particularly since the introduction of IT into general practice in Australia in 1999 [14]. Nonetheless, greater integration of IT into general practice and allied health care providers is still needed [15, 16].

The CDM-Net project was a collaborative project developed and evaluated during 2007–2009 involving 12 Australian and international organisations including an external provider, Precedence Health Care. There were two parts to the project: first the development and formative evaluation of the Chronic Disease Management System (CDMS) to facilitate GPs' use of GPMPs and TCAs and collaboration between GPs, and other health professionals; the second, to evaluate the clinical utility and health professionals' acceptance of the system. The latter is described in this paper.

During the project there was no charge to the GPs for the use of the CDMS. At the completion of the project, a business arrangement was proposed by Precedence Health Care for the participating GPS to consider and/or take up for future use of CDMS.

CDMS is a secure broadband-based web-based interactive software service that complies with Commonwealth and State requirements and the National eHealth Transition Authority standards and guidelines. CDMS interfaces to electronic health records, e-referral and messaging services, and hospitals. The communications infrastructure required by CDMS is provided by existing public broadband networks and provides three innovative services:

(1) continuous, real-time chronic disease care surveillance,

(2) a mechanism to track process (*GPMPs, TCAs, medications, and investigations*) 

(3) an automated open broadband infrastructure [1].

The system delivers collaborative chronic disease management via the Internet utilising electronic applications including:

- remote monitoring; patients upload information such as blood glucose results to the Internet or the health professionals's computer,
- email communication among GPs, other health professionals, and patients,
- Internet-delivered health education information,
- interactive electronic medical records, available to both health professionals and patients [1].

During the planning and development process for GPMPs and TCAs, relevant patient information is encrypted using a public key infrastructure certificate installed on GPs' computers and sent electronically to the external provider Precedence Health Care. Once registered as a CDMS user, users are provided with a personalised secure log-in and password to enable access to the CDMS website [1].

When using CDMS, the process commences when a GP assesses a patient and forwards a referral to Precedence Health Care. All steps are completed electronically; CDMS creates a GPMP which is returned to the GP for approval. Once approved and returned to Precedence Health Care, the option to create a TCA can be enabled by ticking a box. The GPMP is then forwarded electronically to allied health professionals who are generally known to the GP for agreement to participate. For this work, eligible allied health professionals can claim payment using Medicare Items 10950 to 10970, or are paid by the patients for services. The relevant documentation is electronically generated, signed, and distributed to the care team and patient. CDMS tracks the GPMP and TCA and patient outcomes such as medications, biomedical measurements, and appointments. When reviews are due, CDMS automatically generates and electronically forwards an alert to the GP, summarises the relevant information, and automatically creates a draft GPMP review for the GP to approve [1].

## 2. Methodology

### 2.1. Aim

1. To measure the uptake and adherence to CDMS GPMPs and TCAs, clinical health, quality of life indicators, and patients' biomedical parameters.
2. To test the broadband-based network (CDMS) in general practice in the Barwon South West Region Victoria.
3. To assess the adherence of both health providers and patients to GPMPs and TCAs generated through CDMS.

### 2.2. Method

A single cohort before and after study, initially with a nine-month intervention period, with two participant groups: GPs and patients with T2DM. The study period was extended with an additional 6 months, resulting in a fourth data collection after the initial nine-month intervention period.

#### 2.2.1. Recruitment

GP recruitment commenced in July 2008 and ceased at the end of January 2009.

Patients were recruited by their GPs from August 2008 and ceased in February 2009.

#### 2.2.2. GPs—Inclusion Criteria

1. Agree to participate in and support the study and attend three workshops.
2. Have either *Medical Director 3* or *Best Practice* medical software.

#### 2.2.3. Patient Inclusion Criteria

1. Diagnosis of type 2 diabetes.
2. Age range 18–75 years.
3. Access and ability to use a mobile phone, the Internet, or a landline telephone.
4. Living independently.
5. Provide informed consent to share their health information electronically with Precedence Health Care and the interdisciplinary care team.

#### 2.2.4. Patient Exclusion Criteria

1. Pregnant women.
2. Diagnosis of HIV/AIDS.

#### 2.2.5. Data Collection

The majority of data were collected at three time points during eleven months; Time 1: at the commencement of the intervention period, Time 2: approximately half way between Time 1 and Time 3, and Time 3: at the completion of the intervention period (T3). A fourth dataset was collected after the intervention period, during the 6-month extension to the project, and it included metabolic parameters.

GP data were collected using field notes, information compiled at three interactive information workshops, and individual interviews.

Patient data were collected using (a) questionnaires, including the Kessler Psychological Distress Scale-10, and the Control Preference Scale at Time1 and Time 3; (b) CDMS-generated GPMPs and TCAs at Times 1, 2, 3 and 4; (c) Barwon Health.

Outcome indicators included the following:

- the number of GPMPs created
- the number of GPMP reviews conducted (because of the limited study period, only the first 6-monthly reviews could be conducted)
- the number of services carried out as recommended by best-practice guidelines, and the number of investigations completed for the patient cohort:
  (1) biomedical (e.g., HbA_1c_, lipids),
  (2) allied health services,
  (3) home medicines reviews,
  (4) use of services at Barwon Health.

#### 2.2.6. Data Analysis

Quantitative data were analysed using statistical package SPSS v.17. The level of observed effects was determined using a *t*-test. The follow up ratio for GPMPs and TCAs was calculated for “before and after”, but was limited to the “first reviews”.

Qualitative data from the workshops and interviews were analysed using thematic analysis [17]. Data were analysed according to the Framework Method [18] and verified independently by two investigators. When there was a difference of opinion, the investigators discussed the issues to reach agreement [18].

#### 2.2.7. Ethics

Ethics approval was obtained from Barwon Health Research Ethics Advisory Committee and Monash University Standing Committee on Ethics in Research Involving Humans, and noted by the Deakin University Human Research Ethics Committee.

## 3. Results

Twelve GPs from seven general practices and 113 patients agreed to participate. No GPs withdrew during the intervention period; 14 patients did not provide all the required data as the study progressed, hence the final patient data set comprised 99 patients at Time 1, 93 at Time 2, 80 at Time 3 and 80 at Time 4 (Table 1). At Time 1, the 99 cohort comprised 61 males and 38 females with an age range of 31 to 83 years; 83% were Australian born and 64% lived at home with their spouses. For the 99, the duration of their diabetes ranged from a new diagnosis to 34 years, and 78% were registered with the National Diabetes Services Scheme.

### 3.1. Uptake of and Adherence to CDMS GPMPs and TCAs, Clinical Health, Quality of Life Indicators, and Patients' Biomedical Parameters, Medicines Profile, and Use of Barwon Health Services

According to practice management data for the two years prior and the one year after the introduction of CDMS, the number of GPMPs (Item 721) and TCAs (Item 723) claimed by participating GPs during the intervention period increased (Table 2). Because of the limited study period, only the first 6-monthly GPMP (Item 725) and TCA (Item 727) were tracked. Nonetheless, this suggests that the use of CDMS has an influence on increasing the number of followups (Items 725, 727), and also suggests that considerable improvement can still be achieved by most GPs who participated in this study.

#### 3.1.1. Clinical Health

Duration of diabetes diagnosis ranged from a new diagnosis to 34 years, with 78% registered with the National Diabetes Services Scheme. The majority of patients reported self-monitoring their blood glucose. Self-monitoring was associated with a longer duration of diabetes, lower mean HbA_1c_, older age, lower mean cholesterol, having a GPMP prior to the introduction of the broadband-based service, CDMS (Table 3). 

The trend suggests that females were more likely than males to monitor their blood glucose and write their results in a record book.

#### 3.1.2. Quality of Life Indicators

The Kessler Psychological Distress Scale-10 was measured at Times 1, 2, and 3. Overall, low levels of distress were reported. There was very little change in total scale scores for patients over time and gender, but those patients who reported having a disability (*n* = 20) had slightly higher distress scores at each time point than those who did not report a disability (*n* = 71). 

The Control Preference Scale was measured at Times 1 and 3, to assess patient's preferred level of involvement in medical decision-making. Forty-one (41%) patients at Time 1, and 31 (31%) patients at Time 3 indicated they preferred to share responsibility for decision-making with their GP. However, 17 (17%) patients at Time 1 and 21 at Time 3 (26%) indicated they preferred the doctor to make the final decision. There was no significant change in patients' self-reported medical decision-making preference style between Times 1 and 3. 

#### 3.1.3. Patients' Biomedical Markers

Eight metabolic parameters were recorded during the intervention period. Not all patients had measurements recorded at more than one time point, and therefore data are for the subsets of patients. Mean HbA_1c_ decreased from 7.41% (Time 1) to 7.05% (Time 4) (*n* = 23); low-density lipoprotein decreased from 2.24 (Time 1) to 1.96 (Time 4) (*n* = 17); triglycerides from 2.21 (Time 1) to 1.89 (Time 4) (*n* = 19) and total cholesterol from 4.31 (Time 1) to 4.04 (Time 4) (*n* = 19) (Table 4). 

At Time 1, blood pressure was recorded for every patient in the study, HbA_1c_, HDL, LDL, triglycerides, and total cholesterol were recorded for more than half the sample and microalbumin was recorded for just under a third of the sample.

#### 3.1.4. Medicines Profile

Prescribed medicines were recorded for 96 of the 99 participants during the intervention period. Patients were prescribed between zero and 23 medicines at Time 1. Patients aged >66 years were prescribed a greater number of medicines (range 0–23 medicines) compared to patients aged <65 years (range 0–18 medicines). The relationship between the number of prescribed medicines and gender or income was not significant. Duration of diabetes was longer for patients prescribed one or more diabetes medicines (oral hypoglycaemic agents (OHAs), insulin, lipid lowering agent, and antihypertensive agent) than patients not prescribed one of these medicines. There were no changes to recorded prescribed medications or dose regimens at Times 2, 3, or 4. Seventy-one patients were prescribed oral hypoglycaemic agents. Among the 66 patients with HbA_1c_ recorded at Time 4, those prescribed an oral hypoglycaemic agent (*n* = 46) had significantly higher HbA_1c_ than patients not prescribed an oral hypoglycaemic agent (*n* = 20).

#### 3.1.5. Barwon Health Service Use

During the CDMS intervention, six types of services were used by this patient cohort available through Barwon Health:

- hospital admissions,
- allied or community health visits,
- emergency department,
- medical imaging,
- outpatients,
- pathology.

Of the 99 patients, 42 did not use any of the above Barwon Health services, 26 used one service, 13 used two services, two used three services, six used four services, eight used five services and two patients used all six services. Patients aged ≥66 years were more likely to use one or more Barwon Health services than those aged ≤65 years. There were no significant differences in services used between the genders. There was a trend indicating patients who used at least one Barwon Health service had a longer duration of diabetes and lower HbA_1c_ at Time 1 than those who did not use a Barwon Health service (Table 5). 

Patients made between zero and 230 attendances to one or more BH service. Two patients had extremely high numbers of attendances (177 and 230). Excluding the two extreme cases, Barwon Health attendances per patient ranged between zero to 125. 

### 3.2. To Test the Broadband-Based Network (CDMS) in General Practice in the Barwon South West Region Victoria

All participants were asked about the advantages and disadvantages, and their satisfaction of the CDM-Net care planning system. The qualitative results indicated that, while there was positive critical feedback of CDMS in the early development stages, overall feedback about CDMS use and the impact of the system to diabetes management was positive. Most health professionals felt that sharing patients' health information electronically was helpful and made a difference to the care they provided, thus interdisciplinary communication was enhanced. Regarding their satisfaction with the implementation and use of CDMS, health professionals, including GPs, identified advantages and disadvantages. Advantages included improved communication between health professionals, individual providers able to electronically update the GPMP and TCA, saves paperwork, faxing, and time; disadvantages included that the current version of CDMS is not user friendly, it is tedious to update documents, and it is something else to take up a good bit of time. These responses reflected individuals' involvement in the study and are reported more extensively elsewhere.

### 3.3. Assess the Adherence of Both Health Providers and Patients to GPMPs and TCAs Generated through CDMS

Results suggest that CDMS had a strong influence on increasing the follow-up ratios for GPMPs and TCAs, with findings suggesting considerable improvement can still be achieved by most GPs who participated in this study Table 6.

## 4. Discussion

GPs using CDMS endeavoured to overcome some of the major barriers to the uptake and use of GPMPs and TCAs including difficulty finding time to develop and review GPMPs and TCAs, the challenges of interdisciplinary communication, the lack of effective followup and review, and support for patient self-management. In the first instance, GPs reported challenges with CDMs to the research team, who in turn, reported this information to Precedence Health Care. These challenges were usually addressed in the ongoing developmental work that continued throughout the intervention period. CDMS satisfied key technical issues by encrypting patient information using a public key infrastructure certificate installed on GPs' computers. 

While this project only considered the outcomes using GPMPs and TCAs in one chronic disease, T2DM, in one region of Victoria, the results suggest an increase in the number of GPMPs and TCAs developed, increased communication among the health providers, followup and review of GPMPs and TCAs, and the use of guideline-based services associated with T2DM management. Prescribed medicines (type, dose, or dose interval) did not change during the study, which is interesting given the trend towards improved metabolic status during the study [4, 7]. One explanation could be that the level of change in biomedical parameters did not provide sufficient evidence for GPs to consider changing medication. Another reason could be that the change in biomedical parameters could be attributed to lifestyle change, or to participating in this study [19]. Pharmacy attendances decreased during the intervention period but the reason for this is not clear; patients may have changed the frequency and/or location of when/where they collected prescribed medicines. 

Allied health service use increased during the study. While the number of reported diabetes educator attendances increased at T2 during the intervention period, less than 50% of the sample reported attending a diabetes educator. Reasons for this may be that some patients did not require the services of a diabetes educator during the study; alternatively, the relatively low rate of reported diabetes educator attendances could reflect a lack of diabetes educator resources in the Barwon South West Region. During the intervention period, 19 diabetes educators worked in the Barwon South West Region to provide services for the 10,074 individuals diagnosed with diabetes [20]. The average waiting time for an appointment with a diabetes educator was two weeks in the hospital diabetes service but waiting times to access diabetes educators in the community were not available. The allocation of five visits per year to allied health professionals might not be enough [6], especially for high risk patients and those with complex care needs, as well as for women during pregnancy.

The results suggest that information technologies, particularly when focused on improved processes of care, better collaboration, and increased practice productivity, may make a significant difference to GPs' use of GPMPs and TCAs in the treatment of patients diagnosed with a chronic illness.

## 5. Strengths and Limitations of the Study

### 5.1. Strengths

The study was undertaken in a “real-life” clinical setting in which GPs, practice nurses, and allied health professionals were engaged in chronic disease management prior to implementing CDMS. CDMS-generated GPMPs and TCAs contained comprehensive diabetes-related clinical information including metabolic parameters, prescribed medicines, and medical complications. 

### 5.2. Limitations

Without a comparison group, it is not clear whether changes in allied health service use were due to GPMP and/or TCA use, to CDMS or to other unrelated factors. Low recruitment to the CDM-Net (research) component may have been due to GPs not having the appropriate software, a reluctance to change from a paper base system to a broad-based service, or that practice staff had expressed concern about their ongoing employment if the CDMS system was utilised in the practice. 

More research that compares CDMS GPMPs and TCAs to GPMPs and TCAs developed not using CDMs is necessary to evaluate the impact of CDMS on allied health service use. 

Replication or continuation of the study is desirable to enable the impact of CDMS on diabetes outcomes to be determined. In particular, the study should include GPs and patients from other divisions of general practice, and female and solo GPs.

## 6. Conclusions

CDMS was associated with small, nonsignificant reduction in lipid levels (low-density lipoprotein, triglycerides, and total cholesterol) and mean HbA_1c_. 

There was no change to recorded prescribed medicines or quality of life, but the use of allied health services appeared to increase during the intervention period. Despite an initial lack of satisfaction with certain aspects of CDMS's functionality identified by allied health professionals, overall satisfaction with CDMS was high among GPs, allied health professionals, and patients. 

The utility of IT-enabled interventions may lie in their ability to facilitate communication among health care providers, and between health care providers and patients. Further research is needed to delineate the contribution of CDMS and IT-enabled interventions to chronic disease management within the Australian context of GPMPs and TCAs.

##  Conflict of Interests

 The authors have no conflicts to declare.

## Figures and Tables

**Table 1:** The number of patients in the study at each time point, the number of patients included in the final analysis, and the percentage of participants retained from Time 1 to Time 3.

	Time 1	Time 2	Time 3	% retained	% retained
				Time 1 to Time 2	Time 1 to Time 3
Number of patients who completed the questionnaire	113	107	94	95%	83%
Number of patients included in the final analysis	99	93	80	94%	81%

**Table 2:** The number of MBS CDM Items for all chronic diseases claimed by GPs in the 2-year period prior to, and 12 months after implementing CDMS (data not provided by all participating GPs).

GP	Prior to CDMS–2007/2008 (from practice data)				After CDMS–2008/2009 (from practice data)			
	721	723	725	727	721	723	725	727
1	7	0	7	0	9	5	9	4
2	—	—	—	—	—	—	—	—
3	25	8	25	0	21	3	22	0
4	17	17	19	17	28	28	36	35
5	14	2	7	3	16	3	19	5
6	6	3	4	—	19	15	7	—
7	3	0	0	0	0	0	0	0
8	9	5	7	1	—	4	17	1
9	24	35	22	54	26	29	26	20
10	—	—	—	—	—	—	—	—
11	3	10	7	8	10	9	7	8
12	63	45	3	7	45	23	0	14

**Table 3:** Patient Self-reported blood glucose and blood pressure monitoring practices during the intervention period (*n* = 99).

Item (*n* patients who responded to each item)	Yes (%)	No (%)	Unsure (%)
Measure blood glucose with a glucose meter (*n* = 99)	74 (75)	24 (24)	1 (1)
Measure blood pressure (*n* = 98)	19 (19)	79 (80)	—
Record BG and BP test results in a record book (*n* = 91)	39 (39)	52 (53)	—
Download BG test results to a personal computer (*n* = 96)	3 (3)	92 (93)	1 (1)

**Table 4:** The number of patients at each time point who had metabolic parameters recorded (*n* = 99).

Metabolic parameter	Time 1 *n* (%)	Time 2 *n* (%)	Time 3 *n* (%)	Time 4 *n* (%)
HbA_1c_	50 (51%)	6 (6%)	15 (15%)	23 (23%)
Microalbumin	31 (31%)	2 (2%)	3 (3%)	13 (13%)
Systolic blood pressure	99 (100%)	13 (13%)	15 (15%)	35 (35%)
Diastolic blood pressure	99 (100%)	13 (13%)	15 (15%)	35 (35%)
High-density lipoprotein (HDL)	53 (54%)	6 (6%)	10 (10%)	15 (15%)
Low-density lipoprotein (LDL)	52 (53%)	5 (5%)	10 (10%)	15 (15%)
Triglycerides	53 (54%)	6 (6%)	9 (9%)	16 (16%)
Total cholesterol	55 (56%)	6 (6%)	10 (10%)	16 (16%)

**Table 5:** The characteristics of patients who used at least one Barwon Health service (*n* = 99).

Characteristic	Patients who used at least one BH service (*n* = 57/99 = 57%)	Patients who did not use any BH services (*n* = 42/99 = 42%)
Male	61.4	61.9
Female	38.6	38.1
Percent reporting they self-monitor blood glucose	73.7	76.2
Mean age (years)	67.81	61.74
Mean duration of diabetes (years)	8.23	7.46
Mean weight (kg)	91.51	91.45
Mean body mass index (kg/m^2^)	31.96	32.53
Mean HbA_1c_ (%)	7.08	8.65

**Table 6:** Patients' attendances at five categories of health professionals within the last three months, including the range and duration of attendances reported at Time 1 (*n* = 99), Time 2 (*n* = 93), and Time 3 (*n* = 80).

	Patients who attended (%)			Range of attendances per patient		
Health Professional	Time 1	Time 2	Time 3	Time 1	Time 2	Time 3
Pharmacist	90 (91)	74 (80)	76 (95)	0–40	0–12	0–24
Podiatrist	39 (39)	51 (55)	42 (53)	0–6	0–6	0–3
Optometrist	34 (34)	28 (30)	30 (38)	0–5	0–10	0–5
Diabetes educator	18 (18)	31 (33)	17 (21)	0–5	0–12	0–2
Dietitian	9 (9)	26 (28)	23 (29)	0–3	0–3	0–6

## Abbreviations

CDM-Net: The Chronic Disease Management Network project
CDMS: Chronic Disease Management System
GPs: General practitioners
GPMP: General Practitioner Management Plan
IT: Information technology
PHC: Precedence Health Care
T2DM: Type 2 diabetes mellitus
TCA: Team Care Arrangement.
